# Mesenchymal Stem Cells as a Promising Cell Source for Integration in Novel In Vitro Models

**DOI:** 10.3390/biom10091306

**Published:** 2020-09-10

**Authors:** Ann-Kristin Afflerbach, Mark D. Kiri, Tahir Detinis, Ben M. Maoz

**Affiliations:** 1 Department of Biomedical Engineering, Tel Aviv University, Tel Aviv 6997801, Israel; ak.afflerbach@gmail.com (A.-K.A.); mark.kirichenko@gmail.com (M.D.K.); tahirdetinis@mail.tau.ac.il (T.D.); 2 Faculty of Biosciences, Universität Heidelberg, 69120 Heidelberg, Germany; 3 Sagol School of Neuroscience, Tel Aviv University, Tel Aviv 6997801, Israel; 4 The Center for Nanoscience and Nanotechnology, Tel Aviv University, Tel Aviv 6997801, Israel

**Keywords:** mesenchymal stem cells, in vitro models, microfluidics, organs-on-a-chip, scaffolds, organoids

## Abstract

The human-relevance of an in vitro model is dependent on two main factors—(i) an appropriate human cell source and (ii) a modeling platform that recapitulates human in vivo conditions. Recent years have brought substantial advancements in both these aspects. In particular, mesenchymal stem cells (MSCs) have emerged as a promising cell source, as these cells can differentiate into multiple cell types, yet do not raise the ethical and practical concerns associated with other types of stem cells. In turn, advanced bioengineered in vitro models such as microfluidics, Organs-on-a-Chip, scaffolds, bioprinting and organoids are bringing researchers ever closer to mimicking complex in vivo environments, thereby overcoming some of the limitations of traditional 2D cell cultures. This review covers each of these advancements separately and discusses how the integration of MSCs into novel in vitro platforms may contribute enormously to clinical and fundamental research.

## 1. Introduction

In vitro models are heavily used to investigate biological processes and develop therapeutic strategies. Yet the human-relevance of most in vitro modeling approaches remains quite limited, creating a substantial obstacle to the applicability of these approaches to drug development and the study of human physiology [1,2,3]. The human-relevance of prevalent in vitro models is hindered by two main factors. The first is the cell source [4]—A model is only as good as the cells it comprises and the capacity to obtain effective human cell sources remains highly challenging. Commonly used cell sources include primary cells, cell lines and differentiated cells from either embryonic stem cells (ESCs) or induced pluripotent stem cells (iPSCs). Yet, as will be elaborated in what follows, all these cell sources have drawbacks when used as model systems. The second factor limiting the human-relevance of in vitro models is the over-simplicity of the technologies used [4]. Indeed, the most prevalent in vitro model is the standard 2D petri dish culture, which lacks some fundamental features of the human micro-and macroenvironments, including organ-organ interaction [1], 3D environment [5], external forces and the extracellular microenvironment (extracellular matrix [ECM] and signaling cues) [6].

**Figure 1:**
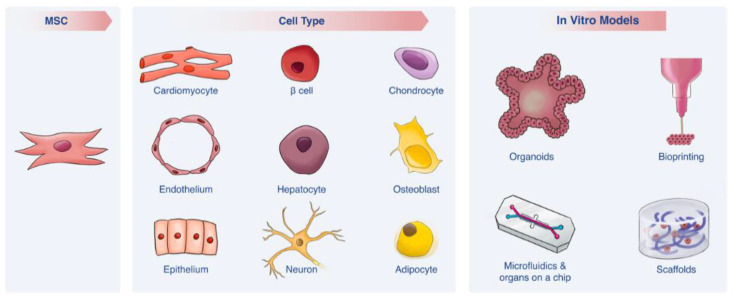
Mesenchymal stem cell (MSC) as a promising cell source for integration in novel in vitro models. MSCs can be differentiated to various of cell types, indicating on its promising potential as a cell source. These potential lineages, as well as MSCs alone, can be integrated with the recent development of novel in vitro tools, such as microfluidics, scaffolds, bioprinting and organoids to enable us providing clinically relevant data, which better mimics the human physiology.

**Figure 2:**
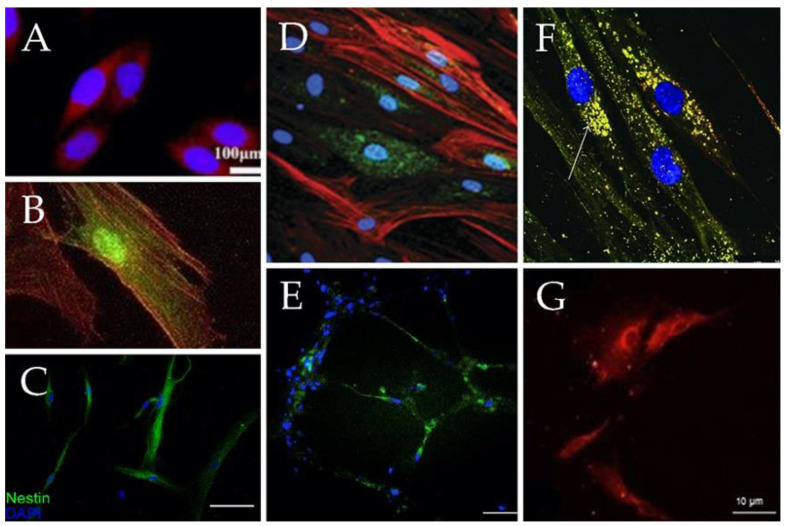
Immunofluorescent staining of MSCs differentiated into different cell types. (**A**) Hepatic differentiation of umbilical cord MSCs confirmed by the expression of hepatocyte-specific gene, cytochrome P450 3A4 (red). Scale bar, 100 µm (adapted from Reference [50]). (**B**) Cardiogenic differentiation of adipose tissue derived MSCs confirmed by the expression of sarcomeric-alpha-actinin (red) (adapted from Reference [34]). (**C**) Expression of Nestin (green) following neural induction of skin derived MSC. Scale bar, 100 µm (adapted from Reference [51]). (**D**) Epithelial differentiation of lung-MSCs after retinoic acid treatment, confirmed by the expression of E-cadherin (green) and anti-smooth muscle actin (red) (adapted from Reference [52]). (**E**) Endothelial differentiation of bone marrow derived MSCs confirmed by the expression of CD31 (green). Scale bar, 1 mm (adapted from Reference [53]). (**F**) Beta cells differentiation of bone marrow derived MSCs confirmed by the co-expression of insulin and c-peptide (yellow). Scale bar, 25 µm (adapted from Reference [54]). (**G**) Epidermal differentiation of umbilical cord MSCs confirmed by the expression of KRT5 (red). Scale bar, 10 µm (adapted from Reference [48]).

Research is continually developing towards overcoming these challenges. With regard to cell source, recent studies have demonstrated the use of mesenchymal stem cells (MSCs) as an alternative human relevant cell source that can be used in engineered platforms recapitulating different human tissues and organs (Table 1; Figure 1 and Figure 2). While MSCs have many advantages over other cell sources, MSC-based in vitro models are still in limited use, perhaps, in part, because of a lack of awareness of their strength. With regard to technology, novel in vitro platforms—such as microfluidic devices and Organs-on-a-Chip, scaffolds and organoids—have emerged to overcome shortcomings of standard 2D cultures [1]. While these technologies have existed for more than a decade, recent developments have made them more robust, easy to use, valid and accessible; indeed some platforms are even commercially available. These advancements have resulted in a new gold standard for studying human physiology in vitro.

This review discusses each of these two developments and the potential synergy between them (Figure 1). Specifically, we first present an overview of MSCs as a promising cell source for in vitro modeling and point out some of the limitations associated with MSC such as yield and lack of functional phenotypes as well as controversial mechanism of action. We then introduce novel in vitro technologies and their individual advantages and disadvantages and explore the possibilities that arise when using MSCs as a cell source in combination with such technologies.

## 2. Mesenchymal Stem Cells as a Cell Source

### 2.1. Brief Research History

MSCs were first identified by Friedenstein and colleagues in the late 1960s. The researchers reported that bone marrow transplantations into kidney capsules of rodents resulted in unexpected bone structure formation in addition to the expected proliferation of bone marrow cells. These observations indicated that bone marrow contained a cell population capable of forming bone structures [7]. However, only in 1991 would the term “mesenchymal stem cells” be introduced to refer to these cells. The term was coined by Caplan, who observed that these cells were able to differentiate into bone, cartilage, tendon, ligament, adipocytes, dermis, muscle, connective tissue and marrow stroma [8]. Another milestone in MSC research was Pittenger’s finding that human bone marrow contains multipotent stem cells that are a subpopulation of stromal cells [9].

### 2.2. Physiological Sources of MSCs

MSCs have their roots in the mesenchyme or mesoderm, the third germ layer in the embryo. During fetal development, these cells migrate to all parts of the body and form muscles, bones, tendons, ligaments and cartilage, as well as bone marrow. The differentiation process and the true origin of the cells still remain somewhat unclear [10].

Unlike ESCs, MSCs can be obtained at all stages of development, through adulthood. Since MSCs can be derived at various ages, it is noteworthy that their characteristics differ according to the age at which the cells were recovered. The sources for retrieval include adipose tissue [11], bone marrow [11], umbilical cord blood [11], dental tissues [12], menstrual blood [13] and skin [14], among many others [15]. The proliferation rate of an MSC culture is highly dependent on the source and donor. For example, MSCs derived from fetal tissue proliferate faster compared with those derived from adult tissue; yet, those derived from adult tissue form a higher number of colony-forming units [10]. MSCs can also be obtained through differentiation of other types of stem cells, namely ESCs and iPSCs. Differentiation protocols and properties of the MSCs were recently nicely reviewed by Abdal Dayem et al. [16]; however, they have some limitations as will be discussed in what follows.

### 2.3. Characteristics

In response to controversy in the scientific community about the nomenclature of MSCs, the International Society for Cellular Therapy (ISCT) published nomenclature guidelines in 2005; a year later, it issued guidelines for the minimal definition criteria. The term “mesenchymal stem cells” remains the most widely used term. However, the term “multipotent mesenchymal stromal cells” is recommended and more appropriate, as the cells’ stemness is not yet fully elucidated [17]. The ISCT proposes the following criteria for defining cells as MSCs—plastic-adherence in culture, multilineage differentiation potential and the presence several markers. The cells must be able to differentiate into osteoblasts, adipocytes and chondroblasts and therefore have the potential to form all tissues these cells form in physiological conditions. The MSCs should express the markers CD73, CD90 and CD105, whereas the markers CD45, CD34, CD14, CD11b, CD79a, CD19 and HLA-DR should be absent [18]. There has not yet been found a marker that is solely MSC-specific, which makes research on the origin and route of differentiation of MSCs somewhat complicated [19].

### 2.4. Applicability of MSCs in In Vitro Models

As noted above, the identification of suitable cell sources constitutes a major challenge in the development of clinically oriented in vitro models. Ideally, cells used in such models should fulfill the following criteria—they should be similar to the original tissue, robust, cost effective, human-relevant, available in large scale and with low variability. However, currently, there is no single cell source that fits all these requirements. For example, rodent primary cells are not human-relevant [20] and the clinical relevance of cell line models has been continuously questioned.

ESCs and iPSCs have been hailed as human cell sources with vast potential for clinical application, yet these sources, too, present challenges. The use of ESCs for deriving cells is limited by ethical considerations and practical issues such as the lack of available embryos, immunocompatibility and the risk of teratoma formation [21]. iPSCs bypass the ethical problems associated with ESCs, yet nevertheless have substantial teratogenic potential, which is a major obstacle to the applicability of these cells in in vitro studies [22].

MSCs offer many of the advantages of ESCs and iPSCs, in that they provide an autologous source of stem cells with the capacity for self-renewal. Crucially, their multi-lineage differentiation potency makes them useful in diverse fields of research. At the same time, MSCs overcome many of the limitations of ESCs and iPSCs, including ethical issues, organ availability and allogenic rejection. MSCs can be derived from various tissues, including adipose tissue, bone marrow, umbilical cord blood and Wharton’s jelly. Their various sources of origin make them readily available to almost any laboratory. Clearly, however, there are also some drawbacks to MSCs, including limited capacity to know how functional the differentiated cells will be. Moreover, differences between cells from different donors may pose a challenge to reproducibility. While MSC are widely used in regenerative medicine, their exact differentiation mechanism, to other cell types is still unclear, which is one of the major limitations of MSCs. This challenge should be considered when using MSCs for both regenerative medicine and in vitro models, as it can significantly affect the interpretation of the results and their reproducibility. For example, more insights on the mechanism of action of MSCs now suggest that they may operate via their secretome to enhance cardiac functionality [23]. Another issue that significantly affected the reputation of MSCs, is the Anversa laboratory case, where they claimed the use of MSC-derived cardiomyocytes for the regeneration of heart muscle. Unfortunately, failure to reproduce these findings has led many to question the validity and reliability of these results and the use of MSC-derived cardiomyocytes became unpopular [24].

Recent studies have further highlighted MSCs’ rich potential as a cell source in revealing these cells’ capacity to differentiate not only into the “classic” MSC cell lineages reported in dozens of studies (e.g., chondrocytes, osteoblasts and adipocytes) [9,25,26,27] but also into additional cell types, including the following (Figure 2)—hepatocytes [28,29,30] (Figure 2A), cardiomyocytes [31,32,33,34] (Figure 2B), neurons [35,36,37,38] (Figure 2C), epithelium [39,40] (Figure 2D), endothelial cells [41,42,43] (Figure 2E), pancreatic β cells [44,45,46] (Figure 2F) and epidermis [47,48,49] (Figure 2G). It remains a challenge to develop methods that elicit MSC differentiation into other cell types such as gut, lung and microglial cells. In what follows we briefly review the current state of the art with regard to MSC differentiation capacity and subsequently elaborate on techniques and materials that have been developed to enhance MSC differentiation into different lineages.

#### 2.4.1. MSC Differentiation Capacity

The various differentiation lineages of MSCs are summarized in Table 1. The duration of a differentiation protocol is highly dependent on designated cell type and ranges from 7 days [55] to 20 weeks.

Banas et al. [28] examined the potential of MSCs to differentiate into hepatic cells in vitro (Figure 2A). The researchers used adipose tissue–derived MSCs from patients of different ages. They showed that with the addition of specific growth factors (hepatocyte growth factor [HGF], fibroblast growth factor [FGF1], FGF4), the cells exhibited several liver-specific markers and functions, such as albumin production, low-density lipoprotein uptake and ammonia detoxification.

In 2017, Szaraz et al. [32] were able to differentiate MSCs into cardiomyocyte-like contracting cells (Figure 2B). Using first-trimester human umbilical cord perivascular cells, which are a rich source of MSCs, the researchers achieved increased cardiomyogenic differentiation, as indicated by elevated expression of cardiomyocyte markers (i.e., myocyte enhancer factor 2C, cardiac troponin T, heavy chain cardiac myosin, signal regulatory protein α and connexin 43) and generation of contracting cell clusters within one week of co-culture on cardiac feeder layers.

Takeda and Xu [37] treated MSCs with exosomes derived from neuronal progenitor cells for one week and observed development of neuronal morphology (Figure 2C) and an elevation in the expression of neuronal markers (e.g., microtubule-associated protein 2 [MAP2] and neuron-specific enolase [NSE]).

In order to differentiate MSCs into keratinocytes (Figure 2D), dos Santos et al. [48] used MSCs withdrawn from the umbilical cord and cultured them in a defined keratinocyte serum-free medium (KSFM) supplemented with epidermal growth factor (EGF) and calcium chloride ions. They evaluated the expression of epidermal markers such as—p63, involucrin and cytokeratins (KRTs) KRT5, KRT10 and KRT14 for 23 days and observed high activity of kallikreins (KLK). KLK are serine proteases that are involved in proteolytic cleavage of corneodesmosomes, an essential event for desquamation.

Janeczek Portalska et al. [42] used growth supplements together with shear force followed by a matrigel assay to differentiate MSCs into endothelial cells (ECs) (Figure 2E). They showed that endothelial-like MSCs were able to take up acetylated low-density lipoprotein (one of the characteristics of ECs) and to create capillary-like structures (which were more stable than the ones formed by human umbilical vein endothelial cells [HUVEC]).

Differentiation of β cells (Figure 2F) from MSCs involves two main steps. First, cells are differentiated into pancreatic progenitors, followed by β cell maturation. This differentiation is mostly achieved by using nicotinamide as indicated in a study conducted by Chen et al. [44]. In this study, the researchers managed to differentiate MSCs into functioning β cells, with a typical morphology (Figure 2f) of isle-like clusters and insulin excretion.

Păunescu et al. [39] cultured MSCs with different growth factors (epidermal, keratinocyte, hepatocyte and insulin-like growth Factor-II) and were able to differentiate the cells into functional epithelial-like cells (Figure 2G). Upon differentiation, these cells acquired a rounded or polygonal shape of epithelial-like cells and expressed epithelial markers such as cytokeratin 18 and cytokeratin 19.

#### 2.4.2. Enhancing MSC Differentiation with Scaffold Techniques

Prior research has revealed that elasticity of a culture substrate affects the differentiation lineages of MSCs [79]. Since this discovery, intensive research efforts have been devoted to engineering various scaffolds for culturing MSCs. Such scaffolds serve both to enhance MSC differentiation protocols and to improve cellular functionality.

Advancements in scaffold design include combinations of different biomaterials [80,81,82], manipulation of the scaffold structures (e.g., including microcarriers) [83] and porous architecture [84]. The benefits of scaffold-based differentiation to classic lineages of MSCs (chondrocytes, osteoblasts, adipocytes) have been widely studied [80,81,83,84,85,86,87,88,89,90,91]. Accordingly, in what follows we will focus on the use of scaffolds for enhancing differentiation into “non-classic” cell types. All the lineages are summarized in Table 2.

Hepatocytes—By growing human MSCs on nanofibrous poly caprolactone (PCL) and collagen scaffolds, the cells were differentiated to hepatocyte-like cells and stayed metabolically active for up to 21 days [92]. The MSC-derived cells on the scaffold showed higher production levels of albumin, urea and transferrin than did the same cells in a 2D culture. In another study, also based on a collagen scaffold, the collagen was cross-linked with heparin, a well-known glycosaminoglycan of the ECM [93]. This process heparinized the gel, which enhanced the cells’ differentiation, viability and functionality.

Neurons—It is known that surface nano-topography of a scaffold enhances the differentiation of stem cells [107] and in particular of neuronal stem cells [108]. Researchers applied this knowledge to the neuronal trans-differentiation pathway of MSCs as well. Yim et al. showed that when MSCs were cultured only on polydimethylsiloxane (PDMS) nanogratings of 350 nm width, the cells were elongated to neuron-like morphology, which induced upregulation of neuronal markers [38]. The authors compared the effect of nano-topography to the effect of retinoic acid (RA) alone and a synergic effect of both nanopattern and RA inductions. They showed that the effect of nano-topography could be even stronger than the classic biochemical induction and were able to grow the cells until day 14.

Gu et al. seeded MSCs on a 3D-cellulosic hydrogel scaffold. The cells were first treated with hEGF and bGFG, later replaced with brain-derived neurotrophic factor (BDNF) and RA. The cells first started to show neuron features after 14 days [94].

**Figure 3:**
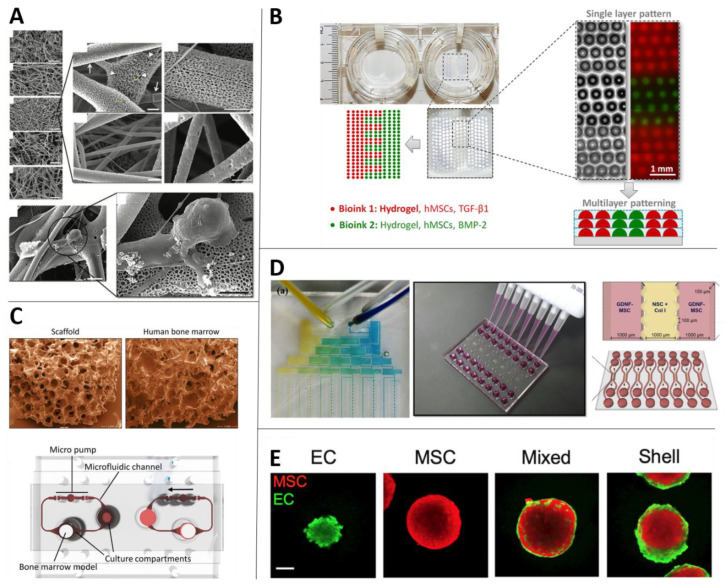
Novel in vitro technologies using MSCs. (**A**) Differentiation of MSCs towards neurons using scaffolds, with different PLA concentrations (adapted from Reference [37]). (**B**) 3D bioprinted MSC patterns in hydrogels (adapted from Reference [109]). (**C**) MSCs in bone marrow scaffolds on OoC (adapted from Reference [110]). (**D**) left: Microfluidic platform to serially dilute morphogens for MSC culture units (adapted from Reference [111]); right: Microfluidic solution for co-culture of MSCs and NSCs to mimic paracrine signaling (adapted from Reference [112]). (**E**) Spatial localization of MSCs and ECs in spheroid co-cultures (adapted from Reference [113]).

Ghorbani et al. worked with an electrically conductive scaffold made of polylactic acid (PLA) with carbon nanotubes and coated with alginate, gelatin and carbon nanotubes [36]. MSCs were cultured on the scaffold and incubated up to 21 days treated only with DMEM and valproic acid (1mM) without any growth factors (Figure 3A).

Epidermis—MSCs are well known as a supportive cell population that is involved in skin regeneration in vivo [114]. Nevertheless, several in vitro studies suggest that MSCs enhanced with growth factors do not differentiate to keratinocytes completely [47,49], meaning the MSCs get the morphology of keratinocytes but they do not acquire the whole set of epidermal markers needed to be considered as keratinocytes. Thus, classic 2D cultures might fall short for this lineage.

Ma et al. showed that it is possible to differentiate MSCs to an epidermal lineage by formulating a skin-like environment based on collagen gel [95]. The MSCs were seeded in a co-culture with fibroblasts on the gel and treated with EGF and vitamin D3. When environmental and physical factors were added to the model, epidermal lineage of MSCs was produced.

Very recent work done by M. Li et al. integrated the scaffold approach with a co-culturing technique to create an independent culture of epidermal-like cells [103]. This was done by growing MSCs on a spongy collagen scaffold and cultivated on a transwell with HaCaT cells (epidermal cell-line) on the other side, which enhanced epidermal morphology and markers.

Cardiomyocytes—There are number of approaches to create this lineage, which include—chemicals, cytokines, microRNAs, culture intermediators and more. Unfortunately, the differentiation efficiency is relatively low, which remains one of the main challenges in the field.

Z. Li et al. dealt with this issue and tested the possibility of inducing this trans-differentiation by growing the cells on a thermosensitive hydroxyethyl methacrylate (HEMA) hydrogel [97]. The researchers showed that when MSCs were cultured on hydrogels with different stiffness, the cells developed calcium channels, gap junctions and grew with higher efficiency (shown as cardiac markers and electrophysiological properties of the cells) than they did under chemical induction and co-culturing with cardiomyocytes.

Other scaffolds have been developed to enhance cardiac differentiation of MSCs, such as PCL [98,99,100] and collagen [101,102]; these approaches demonstrated relatively higher yield compared with the methods based on biological factors alone.

β cells—Many scaffolds have been suggested to enhance the differentiation of MSCs into insulin-producing cells. These scaffolds are constructed from various materials—PCL [103], poly vinyl alcohol (PVA) [104], fibrin glue [105] and collagen with hyaluronic acid (HA) [106]. Compared to 2D cultures, all cultures on scaffold showed significantly higher numbers of genes associated with islet and insulin release.

## 3. Novel In Vitro Technologies and Their Potential for Application with MSCs

### 3.1. Limitations of Traditional 2D Cultures

In vitro cell or tissue culture models are useful for the study of the molecular basis of physiological and pathological responses. Yet, many of the model systems that are most commonly used have substantial shortcomings in terms of their capacity to provide clinical insights, as they do not accurately reflect the complexity of the human body [1]. In particular, cultures often fail to simulate the complex cell–cell and cell–matrix interactions that are crucial for regulating cell behavior in vivo [5] and static culture conditions and excessive amounts of nutrients fail to capture the intricate in vivo environment [115]. The 2D monolayer structure of the cell culture model does not recapitulate the complex structure between the cells and multicellular tissue organization and thus cannot mimic tissue function accurately. For example, 2D monolayer cells do not exert force and thus cannot show in vivo dynamics. Moreover, compared with cells in 3D structures, cells in 2D cultures form fewer gap junctions, which are important for cell communication processes, tissue integrity and function. The discrepancies between 2D cell cultures and 3D cell organization directly affect clinically relevant measurements—For example, drugs diffuse faster in 2D cultures than in 3D cultures, where they need to diffuse across several layers of cells to reach their target [4]. Thus, overall, the shortcomings of 2D cell cultures make it challenging to interpret the results of in vitro experiments in terms of their implications for the whole organism or the whole human body.

In recent years, novel in vitro models enhancing cellular properties and providing better representation of the human body have been developed to address the shortcomings of traditional cell culture approaches [3,116]. In this section, we will focus on the most promising and widely used of these techniques—microfluidics—including the Organ-on-a-Chip (OoC); fabricated 3D structures—including scaffolds and bioprinting; and spheroids and organoids. These models are summarized in Table 3. After introducing each model, we will elaborate on its potential for application with MSCs.

### 3.2. Microfluidics and Organs-on-a-Chip

A microfluidic platform has been defined as a platform that provides “a set of fluidic unit operations, which are designed for easy combination within a well-defined fabrication technology.” Such platforms have been suggested to “[pave] a generic and consistent way for miniaturization, integration, automation and parallelization of (bio-)chemical processes” [153] (Figure 3D).

Microfluidic platforms are based on microfabrication techniques that were first developed for the electronics industry in the late 1950s but were quickly adapted for research in chemistry, biochemistry and biology. In the 1980s and 1990s, microfluidic research platforms began to gain popularity with the emergence of designated equipment for the analysis of such systems. This development marked the initiation of the field of the “lab-on-a-chip” [153]. The development of easy fabrication methods—namely, micro- and soft lithography with materials such as PDMS, polycarbonate (PC) and other polymers—increased the accessibility of microfluidic chips and they began to be used in a variety of applications. Currently, microfluidics are applied widely throughout all scientific fields.

The main advantage of microfabricated chips over a traditional lab environment is the miniaturization of the system. Such miniaturization provides the capacity to control all parameters, reduce costs and achieve high throughput [154]; it is also possible to achieve higher sensitivity than that of traditional technologies. In comparison with standard macrofluidic platforms or traditional cell culture, flow in microfluidics can be better characterized and the diffusion of reagents can be better controlled. Moreover, single cells can be serially processed at high speed and the liquid compartments can be smaller than the size of a single cell. All these features are advantageous in research using cells [153].

There are five major categories of microfluidic platforms, distinguished according to how the system propels the liquid used—capillary, pressure-driven, centrifugal, electro-kinetic and acoustic. Capillary systems use lateral flow; an example of such a system is the test strip used in pregnancy tests. Pressure-driven systems transport the liquid with a pressure gradient, most often with a pump, gas expansion or syringes. Electro-kinetic systems use electric fields to influence charges or dipoles, as in the case of the use of electrophoresis to separate molecules. Acoustic microfluidic systems control droplets on a hydrophobic surface surrounded by air with acoustic (shock) waves. Depending on the kind of system, reagents are injected or pre-deposited [153].

In the biological, biochemical and biomedical fields, microfluidic platforms are used for the following applications—(1) biotransformation, including fermentation and biosynthesis of complex molecules; (2) analytics, where precise measurements with little error are needed; (3) cellular assays, which are widely used in the field of drug development and pharmaceutical sciences [153]; (4) large-scale experiments in which up to hundreds of units are combined; (5) novel in vitro models such as OoCs, which can be used to investigate many different biological research questions in an organ-like setting and are discussed in detail in the following subsection.

#### 3.2.1. Organs-on-a-Chip

OoC models are microfluidic human-tissue culture platforms that mimic the functionality of biological organs or tissues by recapitulating multicellular architectures. These platforms were developed in an effort to overcome gaps in the capabilities of traditional in vivo and in vitro approaches by providing an integrated view of complex physiological systems, at a cell-level resolution [155] (Figure 3C). A key strength of OoC platforms is in providing a means of inducing flow on the cells and inducing mechanical (achieved by vacuum pumps) and chemical manipulations on the cells. Currently, OoC platforms serve as a powerful tool in tissue analysis and disease modeling for biological and pharmacological applications [156]. Some OoC platforms are commercially available [157,158] or can be fabricated in one’s lab with different fabrication techniques [159].

The first OoC model was the “Lung-on-a-Chip,” which was developed by the Ingber research group [160]. The Lung-on-a-Chip is composed of a two-layer channel structure separated vertically by a porous and flexible PDMS membrane. On the upper surface primary alveolar cells were cultured, whereas on the lower channel primary human ECs were seeded. Air flow and culture medium were used to recreate the structure of the lung. The system was able to mimic the expansion and contraction movements of the alveolus, reproduced inflammatory reactions and demonstrated an increase in nanoparticle uptake similar to that obtained in animals [161].

The Lung-on-a-Chip paved the way for the development of several other OoCs, such as Liver-on-a-Chip [162], Gut-on-a-Chip [163] and Kidney-on-a-Chip [164]. The OoC field has continued to evolve rapidly (see Sosa-Hernández et al. [1] and Wu et al. [165] for reviews of recent developments). Recent advancements include the development of OoCs with in situ sensors [166] and platforms that allow for the observation of pharmacokinetic processes such as ADME (absorption, distribution, metabolism, excretion) of various drugs and compounds [161,167]. Further developments focus on the construction of “Multi-Organ-on-a-Chip” or “Body-on-a-Chip” systems, which simulate multi–organ interactions by linking multiple OoCs [168]. This approach considers the human body as a complex system, composed of many organs and tissues with multiple physiological roles and interactions.

In recent years, OoC platforms have been used in diverse applications, producing significant scientific discoveries. For example, OoC platforms have enabled researchers to identify previously unknown metabolic coupling in the human neurovascular unit (NVU) [169], study potential treatments for Covid-19 [170], identify microbiome-gut interactions [171], analyze viral replication of the hepatitis B virus [172], model alcohol injury [173], mimic asthma in a “Small Airway-on-a-Chip” model [174], replicate the physiological and mechanical environment of cardiomyocytes [175] and to translate and correlate OoC with clinical data [167]. Still, much room for progress remains; in particular, many fundamental physiological functions cannot yet be readily modeled on chips, including the immune response and the function of the endocrine system [176].

#### 3.2.2. MSCs in Microfluidics and in Organs-on-a-Chip

Stem cells and MSCs in particular, depend on specific criteria in order to differentiate; these criteria include growth factors, transcellular interactions and signaling cues. Microfluidic systems provide precise control over such factors, by enabling researchers to determine the cellular microenvironment’s bio-physio-mechanical properties, biomaterial properties, biochemical properties and fabrication characteristics [177]. This control contributes substantially to researchers’ capacity to study MSCs per se, as well as to incorporate MSCs effectively into model systems (e.g., OoCs). In what follows, we provide a few examples of such applications.

Characterizing the role of mechanical stimulation in MSC differentiation—Physical and mechanical stimulation have significant effects on the osteogenesis of MSCs [178]. In 2007, a microfluidic chip was designed that helps induce osteogenesis in MSCs by applying cyclic pneumatic mechanical stimulation. Notably, the system can be used to apply specific pressure levels and the chip is suited for high-throughput applications. Cell viability is not influenced by the mechanical stimulation; however, the osteogenic differentiation is accelerated in response to it. Thus, this chip suggests that the function of growth factors can be replaced by mechanical stimulation applied to the system [117].

Cell microenvironment—As noted above, factors that are present in the microenvironment of MSCs, have a crucial role in their cell fate and differentiation. These factors include biochemical cues, in addition to interactions with the ECM and with other cells [179]. Studying and isolating the latter interactions can be challenging, as the different cells need to be in close proximity to each other but without direct physical touch. A study by X. Yang et al. in 2017 demonstrated how microfluidic chips can be used to achieve the necessary conditions for studying the MSC microenvironment. The group was interested in identifying interactions between MSCs and liver tumor cells. On the one hand, such interactions can modify the microenvironment, possibly leading to apoptosis of cancer cells; yet, on the other hand, MSCs have been shown to promote migration of cancer cells and thereby facilitate cancer progression. The researchers designed a microfluidic chip in which HepG-2 cells (liver cancer cells) and MSCs were in a no-contact co-culture and used the platform to investigate the role of biophysical factors and the precise addition of biochemical factors in the homing behavior of the MSCs. MSCs showed a clear preference for migrating towards the cancer cells but not towards other cell types. Addition of the growth factor TGF-β increased the speed of migration, whereas a 3D-microstructure proved to be a barrier that inhibited the migration and homing of the MSCs towards the cancer cells [118].

**Figure 4:**
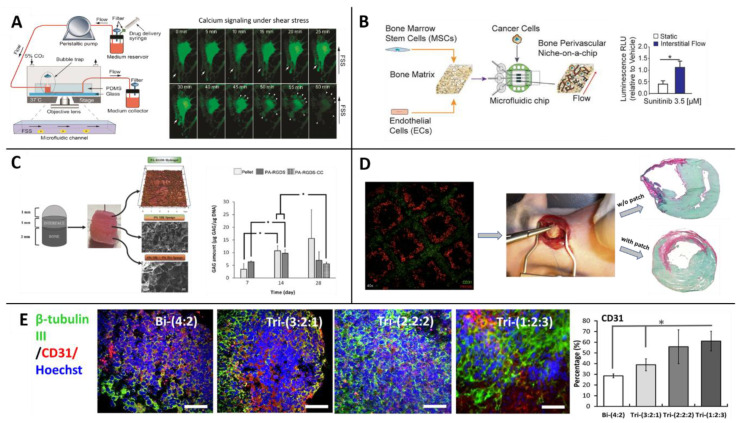
Functional readout of different in vitro models with MSCs. (**A**) Response of MSCs to fluid shear stress shown by calcium signaling in a microfluidic device (from Reference [119]); (**B**) Metastatic colonization on a Bone Marrow-on-a-Chip platform. Treating with a drug (sunitinib) under flow decreased the sensitivity of the treatment to known in vivo values (adapted from Reference [122]); (**C**) Osteochondral bi-layered scaffold model shown increased growth of cells over time (adapted from Reference [129]); (**D**) Bioprinted MSCs/endothelial cells (ECs) cardiac patch improves the regeneration of the heart’s wall after an infract (adapted from Reference [180]). (**E**) Neurovascular spheroids with different neuronal progenitor cells: EC: MSC ratios. Increasing in MSCs amount leads to enhance expression of CD31, which is known as a vascular marker (adapted from Reference [149]).

Morphological studies—Though standard in vitro cultures can be used for morphological studies, the environmental control afforded by microfluidic chips offers substantial advantages. One study, for example, focused on MSCs’ role in vascularization and leveraged a microfluidic platform to shed light on these cells’ behavior in response to different levels of shear stress and pressures of arteries and veins. The researchers placed rat MSCs in a microfluidic device and subjected them to different flow rates and levels of shear stress. The MSCs exhibited contraction and re-spreading in the presence of physiological fluid shear stress [119] (Figure 4A).

Vascularization studies—To create a functional vascular system, it is necessary to obtain cells with a mural phenotype that can build the vessel walls [181]. MSCs can be used to promote angiogenesis and increase the number of network branches. In 2014, Jeon et al. studied the creation of a 3D microvascular network by using MSCs in a microfluidic device in which critical factors determining the acquisition of the mural phenotype could be identified. The microfluidic device enabled MSCs to be co-cultured with HUVECs, resulting in the creation of a physiologically relevant microvascular network. This study also revealed the importance of critical factors such as Ang-1 and TGF-β in the development and differentiation of MSCs towards vascular structures [120].

Controlled release—Microfluidic platforms can be used to control the diffusion of paracrine signals between cells. Recently, microfluidic devices were used to mimic paracrine signaling and to control the diffusion of GDNF secreted by MSCs, leading to the differentiation of neural stem cells into dopaminergic and electro-physiologically active neurons [112].

Mechanism identification—Microfluidic systems can be used to elucidate the developmental mechanisms of MSCs, towards understanding their regenerative mechanisms in bone formation. In a complex microfluidic platform with a serial dilution system and continuous perfusion, bone-marrow-derived MSCs were cultured and condensed to 3D micro-masses. With these micro-masses in the microfluidic chips, effects of different morphogen concentrations could be screened in a high-throughput fashion. It was found that 0.1 ng/µL TGFβ3 results in the maintenance of proliferating MSCs over 7 days and in deposition of collagen II, which indicates chondrocyte differentiation fate [111].

Incorporation of MSCs into OoCs—MSCs were used as a scaffold in a Bone-Marrow-on-a-Chip, alongside cord blood-derived hematopoietic stem cells, to produce a culture of primitive hematopoietic stem and progenitor cells (HSPCs) that survived on the chip for 28 days. The chips contained two independent circular channel systems with two connected culture compartments. The flow was applied with a peristaltic micro-pump that was installed directly on the chip. Within the scaffold, MSCs provided an environment in which HSPCs thrived within 7 days. The niche built by MSCs remained suitable for HSPC culture over the course of the experiment [110].

Another chip system was developed to mimic a more complicated physiological situation where breast cancer metastasis perfuses from a bone to the perivascular niche around blood vessels [122] (Figure 4B). Co-cultures of MSCs:ECs were cultured both in monolayers and in a decellularized bone matrix that was later perfused with breast cancer cells and compared with a static culture. The perfused flow on the tissue led to the formation of dense vascular networks, yet, interestingly the proliferation rate of the cancer cells decreased. The researchers also tested sensitivity to a drug called Sunitinib; the results showed that while the drug inhibited proliferation of the cancer cells in the static culture, it failed to do so in niche under perfusion. (See Section 3.3 and Section 3.4 for additional examples of models capturing the interaction between MSCs and ECs.)

Nelson et al. developed an incorporated chip recapitulating three niches of the bone marrow—a bone layer, central marrow and a perivascular niche [121]. The bone layer was produced through osteogenic differentiation of MSCs and the perivascular niche was produced by seeding MSCs and endothelial cells in the central marrow (a hydrogel network). With this platform, the researchers developed protocols to measure the mobilization of cells and drugs from the bone marrow channel into the peripheral blood. In addition, they tested the damages of ionizing radiation on the bone marrow microenvironment.

#### 3.2.3. Limitations of Microfluidic and OoC Platforms

Microfluidic chips, especially those made out of PDMS, are becoming increasingly prevalent in biological research. PDMS chips provide many benefits, including biocompatibility, transparency and affordability. PDMS also allows for oxygen exchange and the material is generally easy to handle and can be molded into desired shapes [182]. Nevertheless, the use of microfluidic chips presents certain challenges and drawbacks that labs wishing to implement microfluidic devices in general and OoC systems in particular must take into consideration.

The first challenge relates to the need to acquire the appropriate tools and engineering knowledge to fabricate microfluidic devices. While there are companies that offer fabrication services, the chips they produce can be costly and the chips often have to be custom-made to cater to specific research questions.

Moreover, the equipment needed to implement and analyze microfluidic systems, including pumps, heaters, microscopes, gas-supply and connectors, can be expensive and cumbersome. Next-generation microfluidic devices will need to mitigate the need for such bulky equipment.

Additional challenges relate to the chemical properties of PDMS. Specifically, PDMS shows absorption and adsorption of hydrophobic molecules, such as proteins, which can severely disturb cells within the chip. If a chip is being used to model cellular reactions to specific drugs, the applied dosage might eventually differ from the calculated values when the molecules are absorbed or adsorbed by the chip’s material, leading to unknown concentrations and misleading data. Moreover, though PDMS allows for gas exchange, it also allows for evaporation from within the chip, causing problems with the medium supplying nutrients to the cells [182].

Another concern that arises during fabrication of microfluidic devices is the formation of bubbles in the microfluidic channels. These bubbles are difficult to remove and thus can hamper fabrication, control of chips and injure cells [155].

Recent studies have begun to address some of the challenges outlined above. For example, Sung et al. developed on-chip gravity-induced flow that enabled pumpless operation (thereby reducing the need for cumbersome equipment) and prevented formation of bubbles [183]. A key priority in the development of multifluidic and OoC technology is the integration of sensing solutions that can replace off-chip detection methods. Integration of microsensors and nano-sensors will eventually enable researchers to investigate the dynamic responses of the cells to different stimuli and provide information on tissue behavior at a high resolution.

### 3.3. Fabricated 3D Structures: Scaffolds and Bioprinting

Three-dimensional cell cultures are gaining popularity as a means of more faithfully capturing cellular interactions and responses in the natural 3D environment, as compared with 2D approaches. The first steps in culturing cells in 3D included methods such as pellet cultures and micro-mass drops. Briefly, pellet culture refers to growing centrifuged “pelleted” cell spheroids in the bottom of a tube and micro-mass refers to droplets of medium in which cells can grow. Notably, these methods have demonstrated the capacity to enhance differentiation of MSCs to the classic differentiation lineages and even to hepatocytes [184]. Because of their simplicity, pellet cultures are used as a standard in inducing chondrogenesis of MSCs [185]. Nevertheless, pellet cultures have been shown to leave many MSCs undifferentiated or necrotized. Moreover, pellet cultures and micro-mass drops cannot capture multiple compartments of the cellular microenvironment. To address these challenges, more advanced 3D models have been developed, based on scaffold structures and/or incorporating bioprinting to achieve a predetermined cellular organization. We elaborate on each of these aspects in what follows.

#### 3.3.1. Scaffolds

Scaffolds are a broad term for 3D biocompatible structures produced from various biomaterials and are extensively used both in vitro and in vivo due to their biomechanical and physical properties (Figure 3A). The field of 3D scaffolds has advanced rapidly over the last decade. Developments in the materials and methods used for scaffold fabrication have resulted in models that are increasingly durable, biocompatible and representative of the in vivo microenvironment.

##### Materials Used in Scaffolds

The most common scaffold materials are polymeric materials, which can be divided into two categories—synthetic and natural. Synthetic polymers used in scaffolds include polylactic acid (PLA or PDLLA), polyglycolic acid (PGA) and their co-polymers (PGLA), polydioxanone, PCL, PDMS and so forth. Natural polymer scaffolds, in turn, include different types of collagen, elastin, fibrin, glycosaminoglycans (GAGs), HA, gelatin, silk fibroin, alginate and chitin. Synthetic polymers supply a more diverse range of mechanical and biological properties than natural polymers do. Self-assembly, for example, is a feature of natural polymers that is been now used in the growing field of assembling synthetic peptides that can be the base to new biological scaffolds [186]. In addition, natural polymers have a disadvantage of batch-to-batch variation, which can affect experimental reproducibility. Conversely, synthetic polymers are less biocompatible than natural polymers, which are more similar to the native microenvironment.

Hydrogels are also widely used in scaffold models; these are cross-linked polymeric networks that are swollen with water. Hydrogels can also be classified into natural and synthetic materials. Synthetic materials that are commonly used in hydrogels include polyhydroxyethyl methacrylate (poly HEMA) and its derivatives, matrigel, polyethylene glycol (PEG) and PVA.

Scaffolds can also consist of inorganic compounds, ceramic and metallic materials, though these are less commonly used than polymers [187]. For more comprehensive details on the materials and fabrication techniques available for 3D scaffolds, see reviews in refs. [188,189].

##### MSCs in Scaffold Models

In our overview of MSCs, we discussed the substantial advantages of using various scaffolds to enhance MSCs’ multipotent differentiation lineages (Section 2.4.2). Yet scaffolds integrated with MSCs can also serve as model platforms in their own right. MSCs can fulfill two different roles in scaffold models. The first is as a source for a particular cell type of interest, meaning that MSCs can be differentiated into a specific cell type and then studied. Alternatively, MSCs can be integrated as an additional population of cells within the examined tissue, either as a native population or as a population that is recruited to the tissue for different reasons. Examples of scaffold-based models incorporating MSCs include the following:

Hematopoietic bone marrow niche—The first scaffold models of the hematopoietic bone marrow niche were introduced with either MSCs or hematopoietic stem cells (HSCs) alone [91,190,191]. Later, more sophisticated models were introduced, such as co-cultures of HSCs with MSCs in collagen gels [123], PCL, PLGA and fibrin [124] and also PEG hydrogels [125]; and disease models of the bone marrow (e.g., myeloma, leukemia and metastasis of breast and prostate cancers) [126,127].

Osteochondral constructs—Modeling osteochondral tissue requires a biphasic approach—It is necessary to induce MSCs to differentiate into both cartilage and bone lineages within the same model. Therefore, scaffold models intended for this purpose usually consist of a layer structure in which different layers have different compositions and concentrations of biomaterials [128,129,130] (Figure 4C). This type of 3D model is especially important for studying osteochondral defects, such as osteoarthritis, which is a joint disorder characterized by degeneration of the cartilage tissue. Due to the complex structure of the osteochondral complex, Lozito et al. developed a microsystem of the joint complex to use as an in vitro model of the disease. The model contains aspects from both scaffolds and microfluidic systems, connecting the whole system to flow. The different parts of the models are built from tissue-specific ECM components on which MSCs have been cultured. In this design, MSCs fulfill a double role—as native MSCs in the bone and cartilage spaces and maintaining a fibroblastic phenotype in the synovial lining [131].

#### 3.3.2. Bioprinting

A potential benefit offered by 3D in vitro models is the opportunity not only to examine interactions of biomaterials, biomolecules and cells in 3D space but also to capture the significant role of 3D architecture and organization in these interactions. With regular scaffold models, which involve prefabricated scaffold structures, it is not straightforward to control cellular organization. Yet 3D bioprinting—that is, the use of printing technology to deposit biomaterials in a desired 3D arrangement—can be used for this purpose, including fabrication of irregular shapes (Figure 3B).

We note, however, that it is possible to combine bioprinting with other methods; for example, 3D printing technology can be used to produce scaffolds. Compared with molding and porous scaffolds [192], bioprinted scaffolds can provide a higher spatial resolution of structural compartments (biomaterials, biomolecules) and more control over the distribution of cells inside. For detailed reviews of the fast-growing field of 3D bioprinting, see references [193,194].

##### Bioprinting Tissues Containing MSCs

Bioprinting has been used in combination with MSCs to mimic complex 3D structures such as cartilage, bone and tumors. For example, Moore et al. used a CELLINK bioprinter to 3D print a hydrogel scaffold model of breast cancer metastasis in the bone marrow. The model consists of MSCs and breast cancer cells, which are well known for their communication with MSCs during their transition into the bone marrow [132]. In another study, Gurkan et al. used a customized 3D printing method together with MSCs to create multiphase anisotropic cartilage, which mimics the different regions of native cartilage—fibrocartilage (closer to the tendon) and mineralized fibrocartilage (closer to the bone) [109].

##### Bioprinting, MSCs and Blood Vessel Generation

An important novel growing field is 3D bioprinting of blood vessels. As MSCs differentiate into endothelial-like cells and can also be used as a supporting population for differentiation, the combination of vasculature bioprinting with MSCs seems like a promising avenue. In a recent study in canines, Jang et al. incorporated autologous MSCs into small-sized (inner diameter of 4 mm) artificial vessels, which were then integrated into the femoral artery. The authors showed that these vessels were less likely to be rejected by the body compared with vessels printed without MSCs [133].

Prior studies have suggested that MSCs inhibit apoptosis in hypoxic conditions, thereby stimulating angiogenesis [134]. Building on this finding, Gaebel et al. bioprinted MSCs and ECs to create a cardiac patch for cardiac regeneration in rats after myocardial infarction surgery. The researchers found that the cell seeding pattern influenced vessel formation [180] (Figure 4D). Another study showed that MSCs can prevent cell migration of bioprinted ECs on various substrates (e.g., collagen hydrogel), thereby enhancing the capacity to use ECs to create desired microvascular architectures [135].

### 3.4. Spheroids and Organoids

Spheroids and organoids constitute an additional class of 3D models. These models are 3D cultures that do not necessarily grow on supportive scaffold materials and in which cells can grow as an adherent population in a spherical shape that creates its own ECM [195] (Figure 3E). Notably, though researchers sometimes use the terms “spheroid” and “organoid” interchangeably, they are not the same. Whereas spheroids are the more general definition for 3D aggregates of cells, organoids are a sub-category of them and described as more complex clusters, representing mini-organs/tissues or simplified versions thereof. Organoids include micro-anatomy and possess some of the corresponding tissue’s functional capabilities, in addition to as the capacity for self-renewal and self-organization [196,197]. These features constitute a substantial benefit of organoids over simplified spheroids and are advantageous for drug discovery and regenerative medicine [197].

Spheroids and organoids can be used in combination with other in vitro approaches; indeed, previous reviews have discussed the integration of spheroid and organoid cultures with some of the methodologies discussed above, including matrix (scaffold) support [197] and microfluidic platforms [198]. In what follows, however, we focus on spheroid and organoid cultures that are grown independently of such technologies.

Though they provide many benefits over standard 2D in vitro models, spheroids and organoids nevertheless have several limitations. First, because most spheroid and organoid models are not vascularized (a challenge that has been addressed using various techniques, including some of those discussed in this review [199]), they are limited in terms of the diameter their 3D structures can reach [200]. Regarding organoids specifically, another major limitation is that they require long incubation times (up to months and years). In addition, though it is possible to achieve functionality in organoid models, some of them have limited functional readouts. Lastly, most documented organoid cultures lack the organ’s microenvironment [201], including interactions between different cell types such as endothelia and stroma [202]—though organoids that incorporate MSCs, which are at the focus of this section, do include these factors.

#### 3.4.1. MSCs in Spheroids

As noted above (Section 3.3), micro-mass and pellet cultures are considered to be standard methods of 3D culturing of MSCs [185]. Indeed, compared with monolayer cultures of MSCs, construction of MSC spheroids can enhance the cells’ expression of ECM proteins [203,204], in addition to their differentiation into classic cell types [137,138,139], stemness [140,141] and anti-inflammatory properties [142].

Like other types of models discussed herein (see, e.g., Subsection “Bioprinting, MSCs and Blood Vessel Generation” in Section 3.3.2), MSC spheroid models have sought to explore the role of MSCs in the perivascular niche. A recent work by Vorwald et al. on the combination of ECs with MSCs in spheroids was shown to promote the spheroids’ vasculogenic potential [113]. This result addresses the vascularization limitation of organoids in general and shows a good example of how to overcome it. Specifically, to shed light on MSC interactions with the local environment and particularly with ECs.

A primary focus of studies in this vein is to address basic biological questions such as the regulation factors between MSCs and ECs. Studies using MSC-EC co-cultures have revealed, for example, that ECs regulate the MSCs into their osteogenic differentiation and maintaining their quiescence in a 3D cellular context [143]. Studies focusing on the effects of MSCs on ECs and angiogenesis, in turn, have relied both on spheroid co-cultures [144,145] and on spheroids constructed only from MSCs [146,147,148]. These studies were able to improve the therapeutic effect of MSCs on ischemia areas. Spheroid structures create a precondition of hypoxia, thereby “forcing” the cells to upregulate hypoxia-adaptive signals and enhance both angiogenic and anti-apoptotic factors that can later be delivered to a tissue damaged from ischemia. A co-culture model of Shah el al. not only increased those factors but also generated sprout structures that might later be relevant to further angiogenic models [144].

#### 3.4.2. MSCs in Organoids

Only a few studies thus far have integrated MSCs into organoids. Recent work in this area has demonstrated how organoids can serve to capture the tissue microenvironment and cell-cell interactions, towards creating a more complete model of the tissue.

For example, Song et al. synthesized brain organoids to study the interactions between neurons and other cell types (Figure 4E); these interactions had not been previously investigated, though neuronal spheroids corresponding to different parts of the brain had been constructed. The researchers tested tri-cultures of human iPSC-derived cortical neuronal progenitor cells, ECs and MSCs in different ratios. The role of MSCs in these tri-culture systems was both in matrix remodeling and in neurogenesis enhancement [149].

Varzideh et al. produced cardiac organoids in which MSCs served as part of the supporting cell population (together with cardiac progenitor cells and ECs) for differentiating cardiomyocytes. These organoids were able to enhance the maturation of cardiomyocytes towards an adult-like phenotype and develop spontaneous beating [150].

Leeman et al. investigated the differentiation of functioning alveolar progenitor cells as a means of repairing lung injuries. Adding MSCs to 3D progenitor cultures increased differentiation and resulted in the formation of organoid structures. The researchers showed that this effect was caused by MSC-secreted factors that might fulfill the role of stroma in organoid formation [151].

Additional studies have captured the supporting stromal role of MSCs in differentiating hepatic organoids. Takebe et al. created triculture organoids from iPSCs, MSCs and ECs, where the last two acted as stroma for the organogenesis of iPSCs into liver bud organoids. Notably, in addition to contributing positively to processes of differentiation and self-renewal of liver tissue, MSCs’ protective capacity can also enhance the proliferation of tumors [205]. Devarasetty et al. created liver colorectal-tumor organoids consisting of primary human hepatocytes, coloncarcinoma HCT116 and MSCs. They showed that the MSCs created a stroma-like environment, driving rapid formation of tumor organoids. As a result, an unexpected decrease in the sensitivity of the tumor to drug treatment was observed [152]. These results are in line with the anti- inflammatory properties of MSCs and the fact that these properties are enhanced within spheroidal structures [142]. Here, as well as in healthy tissues, MSCs support to the formation of the organoid and improve the model’s capacity to represent the in vivo environment.

## 4. Future Perspectives and Conclusions

MSCs show great potential as a cell source in in vitro models. Their capacity to differentiate into multiple functioning tissues, while overcoming ethical and practical limitations, makes them an attractive alternative to widely used cell sources such as ESCs and iPSCs. MSCs have vast potential for differentiation, particularly in the presence of scaffolds, which can enhance specific MSC properties. The integration of MSCs into novel in vitro technologies—such as microfluidics/OoCs, scaffolds, bioprinting and organoids—can produce state-of-the-art, well-controlled, highly precise and human-relevant model systems for unlimited research applications, including fundamental studies, drug delivery and disease models. However, some limitations of MSCs should be addressed in order to enable high reproducibility and improve functionality of the differentiated cells.

While the novel in vitro systems discussed herein offer considerable advantages over conventionally used technologies, in terms of the capacity to mimic complex structures and replicate physiologically and mechanically relevant environments, they still have some limitations and challenges. These include a need for infrastructure and fabrication knowhow, high costs and long incubation times. We strongly believe, however, that creating more robust protocols for MSC differentiation into new cell types and further adapting novel in vitro models to accommodate these cells and to mitigate the limitations outlined herein, will bring us substantially closer to creating human-relevant in vitro models that are affordable and accessible. Such models have the potential to expedite drug development while reducing the use of animals.

## Figures and Tables

**Table 1:** Differentiation lineages of MSCs induced by growth-factors.

Cell Type	Source of MSCs	Key Differentiation Factors	Markers Expressed	Comments	Ref.
**Chondrocytes**	Bone marrow, Adipose tissue, Natal dental pulp, Placenta, Umbilical Cord, Wharton’s jelly	Transforming growth factor beta (TGF-β), insulin-like growth factor (IGF), Bone morphogenetic proteins (BMP), fibroblasts growth factor (FGF) families and galectines.	Type II collagen, Sox9, ACAN, Col2a1, β-catenin, GAG accumulation.	•Natural differentiation pathway.	[9,27,56,57]
**Osteoblasts**	Bone marrow, Adipose tissue, Natal dental pulp, Placenta, Umbilical Cord, Wharton jelly	Ascorbic acid, β-glycerol phosphate, dexamethasone/vitamin D3 and galectines.	Increase in alkaline phosphatase, calcium accumulation, RUNX2	•Natural differentiation pathway.	[9,57,58,59,60,61,62,63,64,65]
**Adipocytes**	Bone marrow, Adipose tissue, Natal dental pulp, Placenta, Umbilical Cord, Wharton jelly	Dexamethasone, indomethacin, insulin and isobutylmethylxanthin.	PPARγ2, LPL, FABP2, FABP4	•Natural differentiation pathway.	[9,59,64,65,66,67]
**Hepatocytes**	Bone marrow, Adipose tissue, Dental pulp, Placenta, Umbilical Cord, Wharton jelly	dexamethasone, FGFs, bone morphogenetic proteins, hepatocyte growth factor (HGF), epidermal growth factor, oncostatin M and insulin	CYP3A4, CYP1A1, CYP2C9, albumin, CK-18, CK-19 AAT, TAT, heppar antigen	•Characterization analysis of hepatocytes markers is not exclusive for them. Therefore, functional assays are needed to ensure successful differentiation. •Lack of standardization.	[28,50,68,69,70,71,72]
**Cardiomyocytes**	Bone marrow, Adipose tissue, Placenta, Umbilical Cord Blood, Wharton jelly	5-Azacytidine (5-aza), miRNAs, IGF-1, insulin gene enhancer binding protein ISL-1, basic FGF (bFGF), TGF-β family, BMP-2, Caveolin-1, Vanilloid receptor 1 and Histone deacetylase 1.	ANP, cTnT, α-MHC, GATA4, Nkx2.5, CX43	•5-aza considered as a carcinogen. •Some of the inducers have low differentiation efficiency.	[31,34,73,74]
**Neurons**	Bone marrow, Adipose tissue, Dental pulp, Placenta, Skin, Umbilical cord	bFGF, human epithelial growth factor (hEGF), Brain-derived neurotrophic factor (BDNF), all trans retinoic acid (RA). Can be also derived with neuronal progenitors’ exosomes.	Nestin, βIII tubulin, tyrosine hidroxilase, synapthophysin, NURR1, MAP2	•Show robust neuronal electrical activity. •The neuronal functionality of those differentiated cells is controversial.	[35,37,51,64,75,76,77]
**Epithelium**	Bone marrow, Wharton jelly, Lung	hEGF, RA, keratinocyte growth factor, HGF and IGF-II.	CK-18, CK-19, occluding, CD9	•Protocols based on co-cultures cause undesired cell-to-cell interactions.	[39,52,78]
**Endothelial cells**	Bone marrow	Vascular endothelial growth factor (VEGF), bFGF, IGF, EGF, ascorbic acid and heparin.	vWF, VE-cadherin, VEGFR-2	•After differentiation, induced-MSCs successfully created vessel like structures. •More research is needed until applicate it in clinic.	[54]
**Pancreatic β cells**	Bone marrow, Adipose tissue, Urine, Dental, Pancreas, Wharton jelly, Placenta, Umbilical cord, Amniotic fluid	Nicotineamide, L-taurine, sodium butyrate, exedin and glucagon-like peptide-1.	Insulin, glucagon, Glut-2, PDX1, NKX6.1, NEUROD1, NGN3, MAFA	•Differentiated cells can even integrate to pancreatic tissue and become mature. •Less tumorigenic effect than other differentiated cells.	[45]
**Epidermis**	Bone marrow, Umbilical cord	EGF, FGF, Insulin, RA and CaCl_2_, Keratinocyte serum-free medium	P63, CK19, pan-cytokeratin, beta1-integrin, involucrin, KRT5, KRT10, KRT14	•The cells might show epidermal-like morphology but they do not differentiate into keratinocytes.	[47,48,49]

**Table 2:** Enhancing MSC differentiation with scaffold techniques.

Cell Type	Scaffold Type	Ref.
**Chondrocytes**	Silk fibroin (SF), SF/collagen, SF/chitosan/GAGs, PLGA, PLA	[80,81,84,86,88,89]
**Osteoblasts**	Silk fibroin/gelatin, SF/collagen, collagen, PLGA, ECM-based structures	[83,84,86,87,90]
**Hepatocytes**	PCL/collagen, collagen/with heparin	[92,93]
**Neurons**	Nano-grafts from PDMS (with RA), cellulosic hydrogels (with EGF, GFG, BDNF, RA), electrically conductive PLA with alginate, gelatin and carbon nanotubes	[37,38,94]
**Epidermis**	Collagen—as a gel (with EGF and vitamin D3) or as a spongy scaffold (with HaCaT cells)	[95,96]
**Cardiomyocytes**	HEMA hydrogels, PCL, collagen	[97,98,99,100,101,102]
**Pancreatic β cells**	PCL, PVA, fibrin glue, collagen/HA	[103,104,105,106]

**Table 3:** Novel in vitro models.

**Microfluidics and Organs-on-a-Chip**	
**Key features**	Dynamic flow, mechanical and shear stresses, miniaturization, biosensors, organ-organ interaction, precise control of differentiation influences.
**Limitations**	More expensive than the regular 2D culture, unwanted absorption of materials, require expertise and special equipment, not all organs exist.
**General use**	Biotransformation, analytics, cellular assays, large-scale experiments in which many microfluidic units are combined (for high-throughput screening), OoC platforms, organ-organ interaction, observation of pharmacokinetic processes.
**Use with MSCs**	Osteogenesis ([117]), interaction with liver cancer ([118]), vascularization ([119,120]), neuronal differentiation ([112]), bone regeneration ([111]), Bone Marrow-on-a-Chip ([110,121]), perivascular cancer metastasis ([122]).
**Fabricated 3D structures—Scaffolds and Bioprinting**	
**Key features**	3D porous structures, biomechanical forces, ECM mimicking, printing with high spatial accuracy, biodegradability.
**Limitations**	Lack of vascularization and flow, conventional fabrication methods of scaffolds do not provide high accuracy in the microenvironment, large scale biomanufacturing issues, a challenge to incorporate multiple cell types.
**General use**	Enhancing differentiation, diverse complex geometric structures, regenerative medicine, tissue engineering.
**Use with MSCs**	Enhancing differentiation to various lineages (Section 2.4.2), hematopoietic niche in health ([123,124,125]) and disease ([126,127]), osteochondral niche in health ([128,129,130]) and disease ([131]), cancer metastasis in the bone marrow ([132]), multiphase cartilage tissue ([109]), bioprinting blood vessels for implantations ([133,134]) and research ([135,136]).
**Spheroids and Organoids**	
**Key features**	Self-assembly, self ECM fabrication, organoids are defined by functionality of the organ.
**Limitations**	Poor cell viability, long incubation times for some organoid models, not all organs are mimicked, limited functional readouts, usually lack of vascularization and stroma.
**General use**	Organogenesis, drug discovery, regenerative medicine.
**Use with MSCs**	Enhancing properties of differentiation ([137,138,139]), stemness ([140,141]) and immunomodulation ([142]), studying interactions with endothelial cells ([143,144,145,146,147,148]), stromal role in organoids of neurons ([149]), cardiomyocytes ([150]), lung epithelium ([151]) and liver colorectal tumor ([152]).

## Abbreviations

α-MHC	α-myosin heavy chain
5-aza	5-Azacytidine
AAT	alpha-1 anti-trypsin
ACAN	Aggrecan
ANP	trial natriuretic peptide
BDNF	brain-derived neurotrophic factor
BMP	Bone morphogenetic proteins
CK18	cytokeratin 18
CK19	cytokeratin 19
COL2A1	chondrocyte marker type II collagen
cTnT	cardiac troponin T
CX43	connexin 43
ECs	endothelial cells
ESCs	embryonic stem cells
FABP2	Fatty acid-binding protein 2
FABP4	fatty acid binding protein 4
FGF	fibroblasts growth factor
GAG	glycosaminoglycan
GATA4	GATA binding protein 4
HA	hyaluronic acid
hEGF	human epithelial growth factor
HGF	hepatocyte growth factor
HSCSs	hematopoietic stem cells
HSPCs	hematopoietic stem and progenitor cells
HUVEC	human umbilical vein endothelial cells
IGF	insulin-like growth factor
iPSCs	induced pluripotent stem cells
ISCT	International society for cellular therapy
KLK	Kallikreins
LPL	lipoprotein lipase
MAP2	microtubule-associated protein 2
MSCs	mesenchymal stem cells
Nkx2.5	homeobox protein 2.5
NURR1	nuclear receptor related 1 protein
NVU	neurovascular unit
OoC	Organ-on-a-Chip
PC	polycarbonate
PCL	polycaprolactone
PDMS	polydimethylsiloxane
PEG	polyethylene glycol
PGA	polyglycolic acid
PGLA	poly lactic/glycolic acids co-polymers
PLA/PDLLA	polylactic acid
poly HEMA	poly hydroxyethyl methacrylate
PPARγ	peroxisome proliferator-activated receptor γ
PVA	polyvinyl alcohol
RA	retinoic acid
RUNX2	runt-related transcription factor 2
SF	Silk fibroin
TAT	tyrosine amino transferase
TGF-β	transforming growth factor beta
VE-cadherin	vascular endothelial cadherin
VEGF	vascular endothelial growth factor
VEGFR-2	vascular endothelial growth factor receptor 2
vWF	von Willebrand factor
